# Foley’s Catheter as a Simple Adjunct for Membrane Reduction in Late Rescue Cerclage: A Case Report

**DOI:** 10.7759/cureus.105808

**Published:** 2026-03-25

**Authors:** Bandana Bharali, Himangshu Malakar, Anamika Chaudhary, Prateeti Baruah, Prajnya Anubhuti

**Affiliations:** 1 Obstetrics and Gynaecology, All India Institute of Medical Sciences, Guwahati, Assam, IND

**Keywords:** case report, cervical insufficiency, preterm birth, rescue cerclage, shirodkar cerclage

## Abstract

Advanced cervical dilatation with prolapsed membranes in the late second trimester is associated with imminent pregnancy loss or extreme prematurity. Rescue cerclage may improve outcomes in selected patients with intact membranes.

We report a case of a 32-year-old G2A1 (gravida 2, abortion 1) woman at 27+4 weeks of gestation with painless cervical dilatation and bulging membranes. After excluding infection and uterine activity, emergency Shirodkar cerclage was performed under spinal anaesthesia. The prolapsed amniotic sac was reduced using a Foley catheter balloon technique prior to suture placement. The pregnancy was prolonged by six weeks and five days. She later presented with preterm prelabour rupture of membranes at 34+2 weeks and delivered vaginally a healthy male neonate weighing 2287 g following cerclage removal. Both mother and baby were discharged in stable condition.

This case highlights the use of Foley catheter as a low-cost, safe technique during rescue cerclage. Even beyond 27 weeks, careful selection and meticulous surgical technique can achieve meaningful prolongation of pregnancy and a favourable neonatal outcome.

## Introduction

Cervical insufficiency leads to painless cervical dilatation and recurrent mid-trimester losses or preterm birth in the absence of contractions [1]. Emergency cervical cerclage is considered when women present with cervical dilatation and exposed membranes without signs of infection or labour, and aims to prolong gestation and improve neonatal outcomes [2]. The intervention remains controversial partly because of inconsistent evidence on procedural risks (e.g., membrane rupture, infection) and variable clinician practices [3]. Rescue cerclage poses significant technical challenges and is associated with higher risks of rupture of membranes and chorioamnionitis. Various techniques have been proposed to facilitate membrane reduction, including Trendelenburg positioning, amnioreduction, and use of balloon devices.

Recent data suggest that emergency cerclage may reduce adverse neonatal outcomes and increase latency even in advanced cases when infection is excluded and membranes are intact, particularly within the late second-trimester window [4,5]. We describe an emergency rescue cerclage performed at 27+4 weeks using a Foley catheter-assisted membrane reduction method, resulting in delivery at 34+2 weeks and a favourable neonatal outcome.

This case has been reported in accordance with CARE (CAse REports) guidelines [6].

## Case presentation

A 32-year-old gravida 2, abortion 1, presented at 27+4 weeks of gestation with lower abdominal heaviness and increased vaginal discharge for one day. She denied uterine contractions, fever, bleeding, or frank leaking. Her obstetric history included a spontaneous mid-trimester pregnancy loss at 20 weeks, 10 months earlier. There was no history of uterine instrumentation or cervical surgery. The current pregnancy was spontaneously conceived and uneventful until presentation.

**Figure 1:**
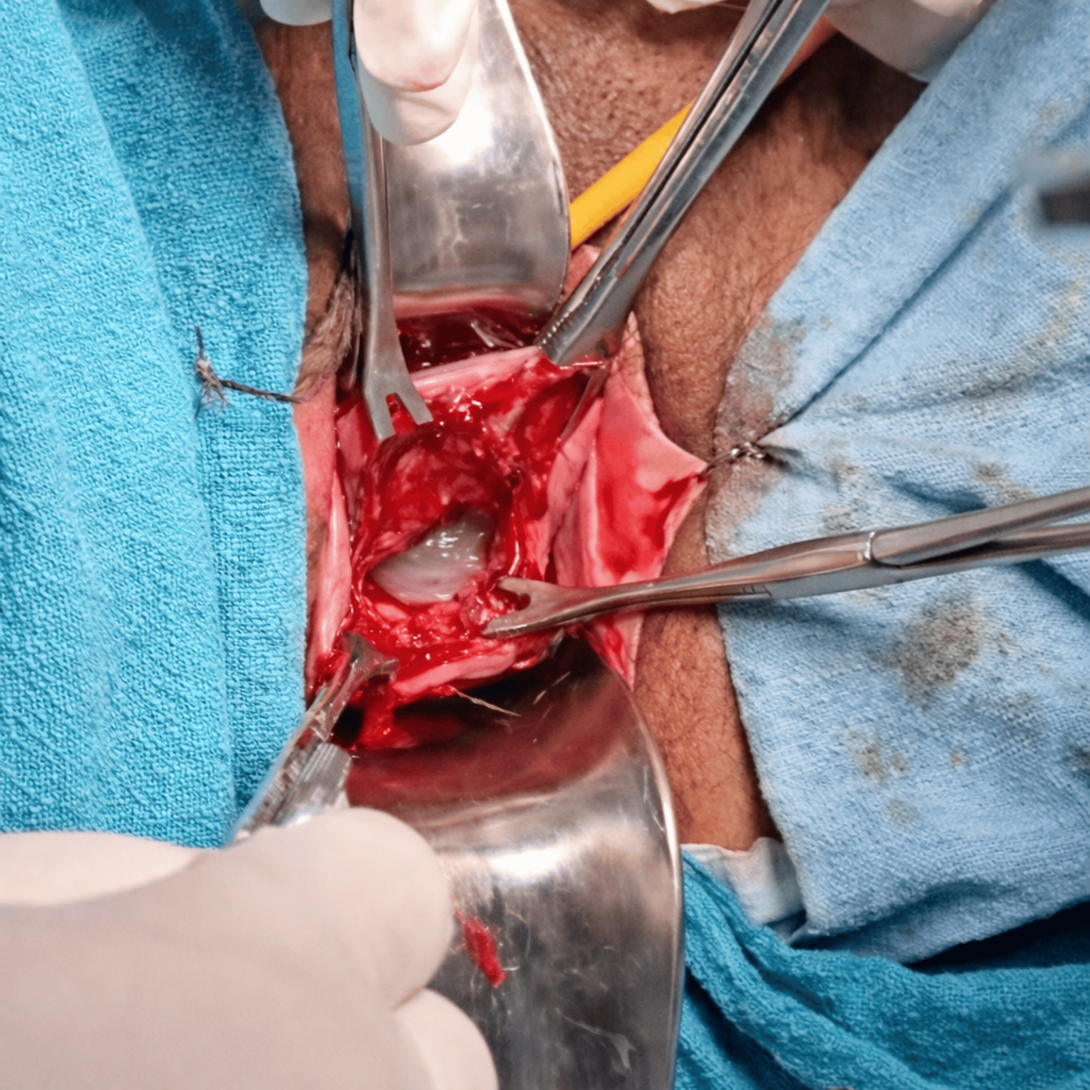
Bulging membranes seen through dilated cervix

On examination, she was afebrile with stable vital signs. Abdominal examination revealed a relaxed uterus appropriate for gestational age and a fetal heart rate of 142 beats per minute. Speculum examination demonstrated a dilated cervix with bulging fetal membranes through the external os (Figure 1). Digital examination was deferred to minimise infection risk.

Transabdominal ultrasound confirmed a live singleton fetus consistent with gestational age and adequate amniotic fluid, with notable cervical shortening and funnelling. Laboratory investigations were as described in Table 1. There was no clinical or laboratory evidence of chorioamnionitis.

**Table 1:** Maternal laboratory parameters prior to emergency cerclage

Parameter	Value	Reference range
Hemoglobin	10.6 g/dl	11-13.9 g/dl
Total leukocyte count	9800/mm^3^	5700-15,000/mm^3^
C-reactive protein	2 mg/L	<5 mg/L

Based on clinical features and ultrasound findings, a diagnosis of cervical insufficiency with prolapsed membranes was made.

**Figure 2:**
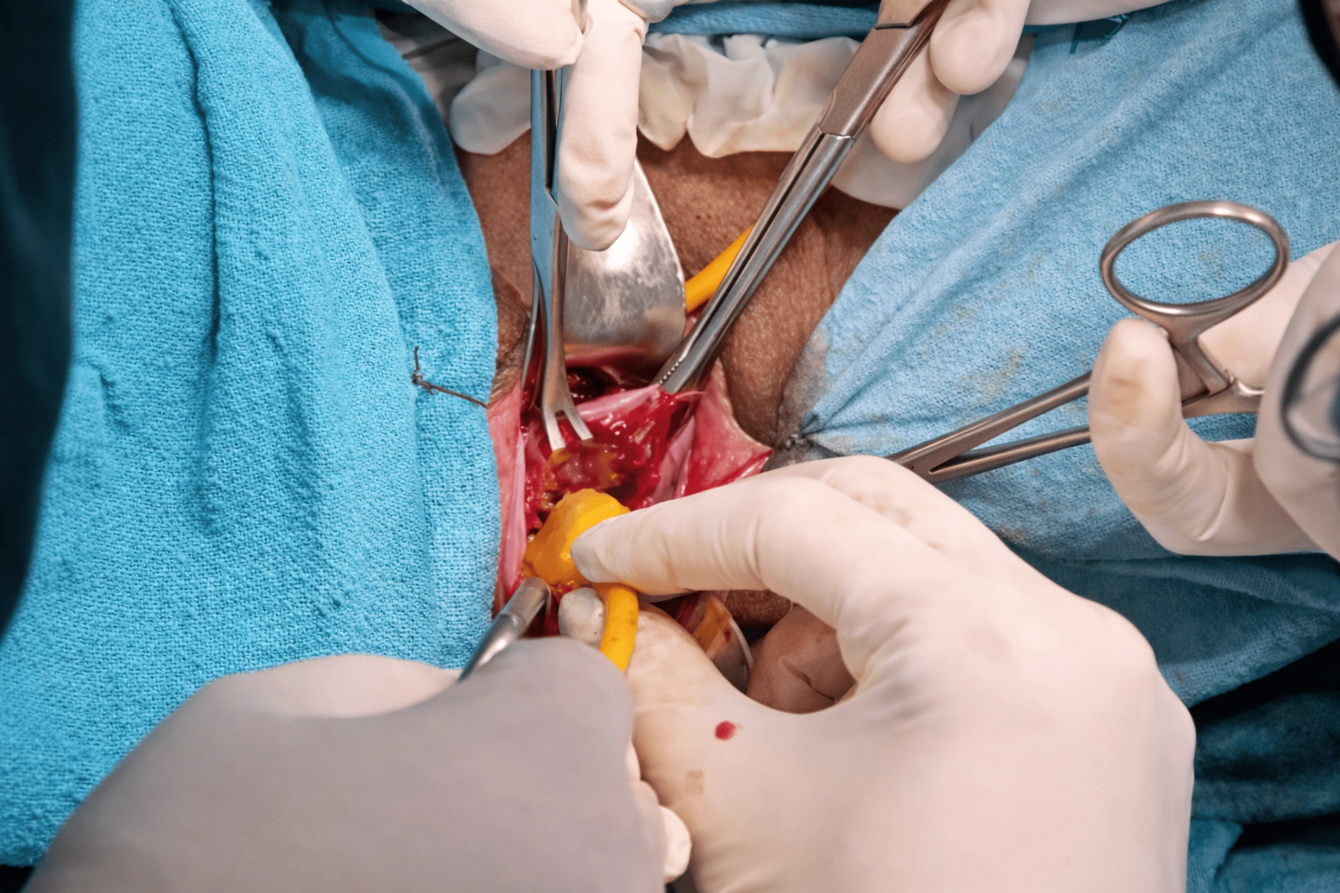
Foley's catheter used to reduce the prolapsed membranes through os

**Figure 3:**
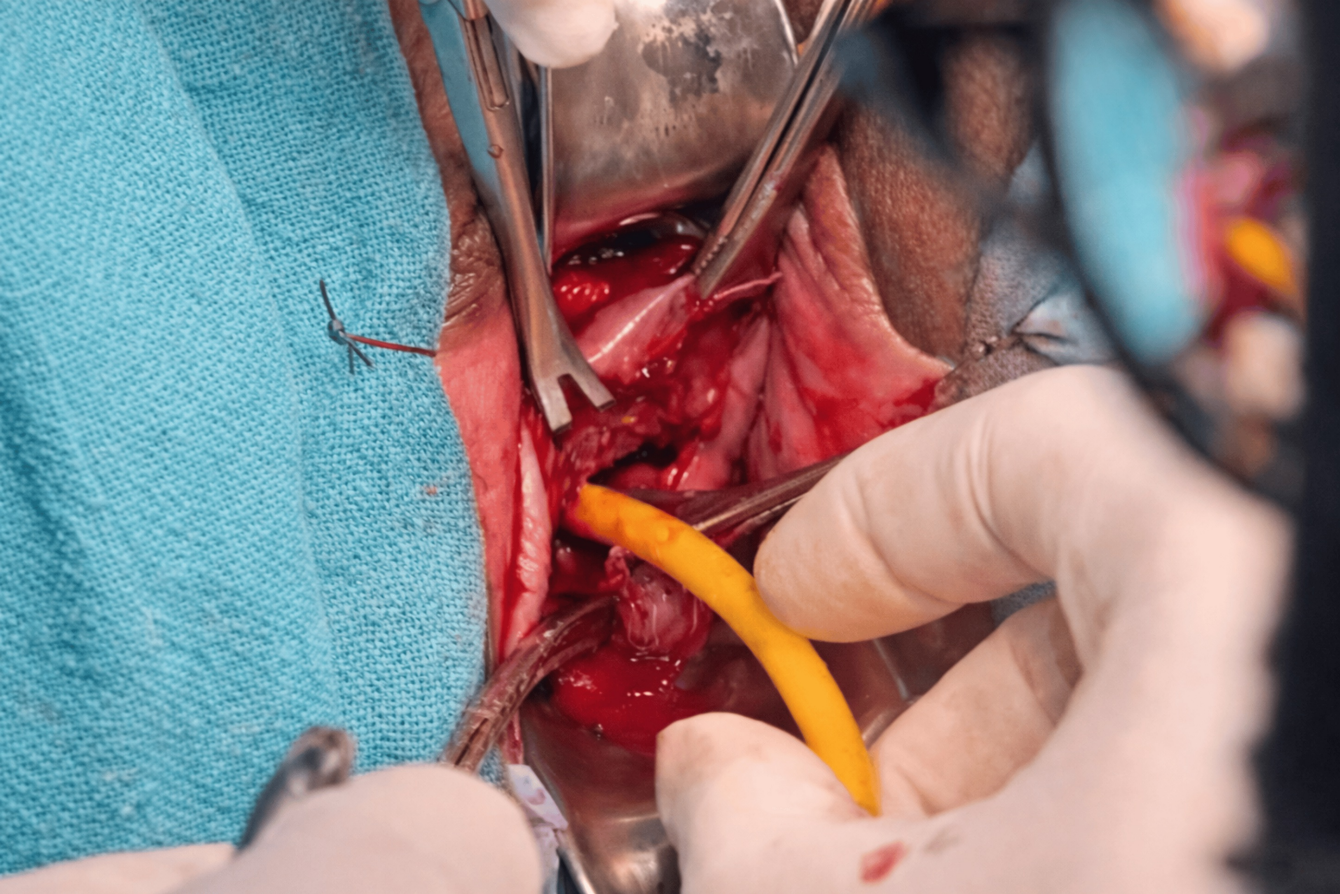
Bulging membranes reduced successfully

**Figure 4:**
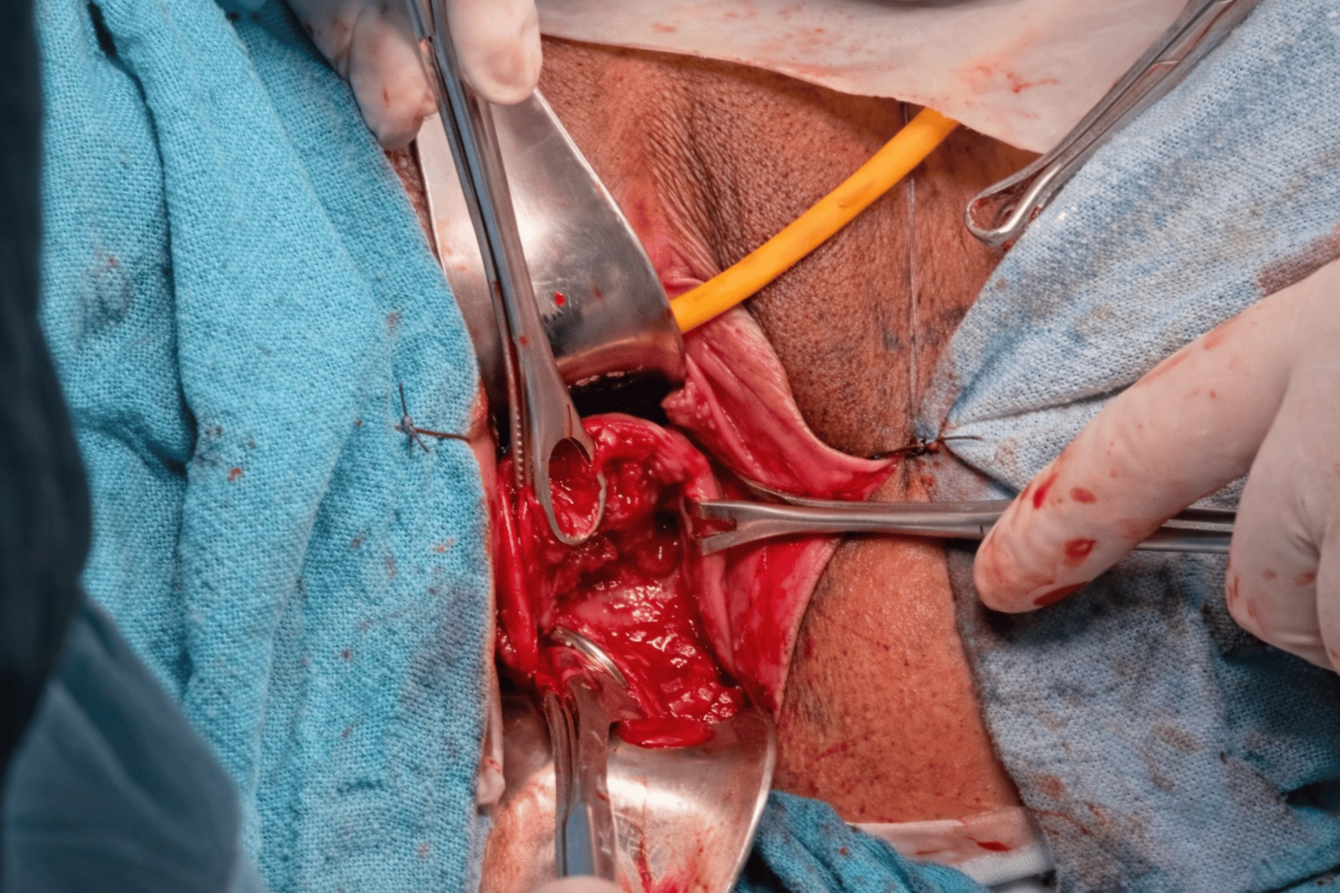
Post-procedure image after successful application of Shirodkar's cerclage

After counselling the patient about the risks, including rupture of membranes, infection, and procedure failure, informed consent was obtained from her for emergency cerclage. Under spinal anaesthesia with the patient in lithotomy and Trendelenburg position, the prolapsed amniotic sac was reduced using a Foley catheter balloon technique, which provides atraumatic membrane repositioning and reduces the risk of rupture before suture placement (Figures 2, 3). Following successful reduction, a Shirodkar cerclage was placed - with dissection of the bladder to allow high suture placement at the internal os. The Foley catheter balloon was deflated and removed after successful suture placement before tightening of the knot (Figure 4).

Postoperatively, she received broad-spectrum antibiotics, short-term tocolysis, and a full course of antenatal corticosteroids for fetal lung maturity. She remained stable and was discharged on postoperative day 5.

Follow-up was conducted fortnightly until 32 weeks and then weekly. Serial clinical assessments and ultrasound scans showed appropriate fetal growth, reassuring Doppler findings, and no signs of infection or cerclage displacement. The pregnancy was successfully prolonged by six weeks and five days.

At 34+2 weeks of gestation, she presented with watery vaginal discharge. Speculum examination confirmed pooling of clear fluid consistent with preterm prelabour rupture of membranes. She was afebrile with stable maternal status and reassuring fetal heart monitoring. The patient progressed to spontaneous labour, and the cerclage was removed, following which she delivered a healthy live male neonate weighing 2287 g with Apgar scores of eight and nine at one and five minutes, respectively. The neonate required short-term observation but did not require ventilatory support. The postpartum course was uncomplicated, and both mother and baby were discharged in stable condition on postnatal day 6.

Both the mother and the baby were found to be doing well at six weeks on a follow-up visit.

## Discussion

Emergency or rescue cerclage continues to generate debate in contemporary obstetric practice due to variability in patient selection, procedural technique, and reported outcomes [3]. While earlier literature expressed concern regarding procedure-related complications such as membrane rupture and ascending infection, more recent systematic evaluations have provided clearer evidence supporting its benefit in appropriately selected patients [4,5]. Meta-analyses demonstrate that, when compared with expectant management, emergency cerclage is associated with significant prolongation of pregnancy and reduced rates of delivery before 28 and 32 weeks in singleton gestations [4]. Furthermore, pooled analyses indicate improved neonatal survival and decreased overall pregnancy loss, although heterogeneity among studies warrants cautious interpretation [5].

Despite this emerging consensus, the gestational age at which rescue cerclage remains beneficial is still debated. Most published data focus on placement prior to 26-28 weeks, reflecting traditional viability thresholds and clinician caution [5]. However, recent cohort analyses evaluating outcomes between 24 and 28 weeks suggest that meaningful latency can still be achieved within this window when infection is excluded and membranes remain intact [7].

Published meta-analyses report that emergency cerclage is associated with an average prolongation of pregnancy ranging between approximately four and seven weeks in carefully selected singleton gestations [4,5]. In the present case, latency following cerclage placement was six weeks and five days, placing the achieved prolongation at the upper end of the reported range. Importantly, this extension allowed advancement from 27+4 weeks - where neonatal morbidity and mortality remain substantial - to 34+2 weeks, a gestational age associated with significantly improved respiratory, neurological, and overall survival outcomes.

Technical considerations are critical determinants of procedural success. Atraumatic reduction of prolapsed membranes is essential to minimise iatrogenic rupture. Balloon-assisted membrane reduction using a Foley catheter has been described as a controlled and reproducible technique that avoids direct digital manipulation and reduces pressure on exposed membranes [8]. Incorporating this method likely contributed to procedural feasibility and avoidance of intraoperative rupture in this case. In addition, high placement of the cerclage using the Shirodkar technique may provide enhanced mechanical support by positioning the suture closer to the internal os, which is particularly advantageous in cases of advanced cervical dilatation [1].

Overall, the present case contributes to growing contemporary evidence that rescue cerclage beyond 27 weeks can still confer meaningful clinical benefit and Foley catheter can be used as a readily available, low-cost and safe adjunct to the procedure. Careful patient selection, meticulous membrane management and structured postoperative surveillance remain central to optimising maternal and neonatal outcomes.

## Conclusions

Emergency Shirodkar cerclage with a Foley catheter-assisted membrane reduction at 27+4 weeks successfully prolonged the pregnancy to 34+2 weeks with a favourable neonatal outcome. Intracervical Foley catheter is a safe, simple, and effective adjunct technique in emergency cervical cerclage with prolapsed membranes. In appropriately selected patients, it facilitates successful suture placement and may significantly prolong pregnancy.

Careful selection, meticulous technique, and vigilant follow-up remain key to success. Rescue cerclage beyond 27 weeks may still offer meaningful gestational prolongation in appropriately selected patients.
